# Effect of Nursing Care Delivery Models on Registered Nurse Outcomes

**DOI:** 10.1177/2377960819869088

**Published:** 2019-08-13

**Authors:** Farinaz Havaei, V. Susan Dahinten, Maura MacPhee

**Affiliations:** 1 School of Nursing, University of British Columbia, Vancouver, BC, Canada

**Keywords:** skill mix, mode of care delivery, team nursing, total patient care, nurse outcomes, work environment

## Abstract

The two key components of models of nursing care delivery are mode of nursing care delivery and skill mix. While mode of nursing care delivery refers to the independent or collaborative work of nurses to provide care to a group of patients, skill mix is defined as direct care nurse classifications. Previous research has typically focused on only one component at a time (mode or skill mix). There exists little research that investigates both components simultaneously. This study examined the effect of mode of nursing care delivery and skill mix on nurse emotional exhaustion and job satisfaction after controlling for nurse demographics, workload factors, and work environment factors. A secondary analysis was done with survey data from 416 British Columbia medical–surgical registered nurses. Data were analyzed using hierarchical multiple regression and moderated regression. Registered nurses in a skill mix with licensed practical nurses reported lower emotional exhaustion when caring for more acute patients compared with those in a skill mix without licensed practical nurses. While mode of nursing care delivery was not related to nurse outcomes, work environment factors were the strongest predictors of both nurse outcomes. Skill mix moderated the relationship between patient acuity and emotional exhaustion. Nurse managers should invest in nurses’ conditions of work environments.

## Introduction

As a result of health human resource shortages, finite health-care budgets, and quality and safety concerns (MacPhee, 2014), models of nursing care delivery have been the target of many redesign initiatives. Two key components of models of nursing care delivery are mode of nursing care delivery (MoNCD) and skill mix (Huber, 2013). First, MoNCD is described as the independent or collaborative work of nurses to provide direct patient care to a group of patients (Havaei, MacPhee, & Dahinten, 2019; Shirey, 2008). The two predominant MoNCDs in most acute care settings are total patient care and team nursing (Havaei et al., 2019; King, Long, & Lisy, 2014). In total patient care, one registered nurse (RN) is mainly responsible for the complete care of a group of patients throughout a shift, whereas a designated team of nursing staff members with various competencies, skill levels, and scopes of practice provide care to a group of patients in team nursing (Duffield, Roche, Diers, Catling‐Paull, & Blay, 2010). Second, skill mix is defined as direct care nurse classifications (Harris & McGillis Hall, 2012). In most acute care settings across British Columbia (BC), there are three key nurse classifications: RNs, licensed practical nurses (LPNs), and nursing care aides (Harris & McGillis Hall, 2012). RNs and LPNs are both self-regulated, which means that their registration or licensure and maintenance of their professional standards of practice are overseen by a regulatory body (Havaei et al., 2019; MacPhee, 2014). However, there are differences in their level of education, competencies, and, subsequently, in their scopes of practice. Compared with RNs, LPNs are allowed to care for more stable and less complex patients. Unlike RNs and LPNs, care aides are not regulated. Care aides typically provide nonnursing supports to regulated nurses (e.g., delivering food trays) (Havaei et al., 2019; MacPhee, 2014). In healthy work environments, skill mix decisions are motivated by creating a match between patient needs and nursing competencies.

To compensate for human resource and financial constraints, MoNCD and skill mix have been the frequent target of redesign initiatives. For example, LPNs have been introduced to some high acuity areas and are expected to provide care to unstable and complex patients in conjunction with RN direction. There has also been a paradigm shift towards a team-based MoNCD that places RNs and LPNs in nursing teams sometimes in absence of a clear understanding of each other’s roles and responsibilities. The impact of these redesign initiatives on nurse outcomes is unclear.

## Review of Literature

Efficient health human resource management requires flexible MoNCDs that consider multiple factors such as the patient population and nursing skill mix (Fernandez, Johnson, Tran, & Miranda, 2012). In one study examining the relationship between nursing skill mix and MoNCDs, researchers found that skill mix was a determinant of MoNCD (Duffield et al., 2010). When comparing team nursing to total patient care, total patient care was associated with a higher proportion of RN hours to all nursing hours and team nursing was associated with a higher proportion of LPN hours to all nursing hours. Because skill mix and MoNCD are related, it is important to study both components simultaneously in order to control one factor while the other factor is examined with respect to nurse outcomes. When MoNCD or skill mix decisions are only efficiency driven, RNs may experience significant stress because of the mismatch between nurse competencies and patient needs that may result from these decisions.

Only one Canadian secondary analysis was found in which researchers examined the effect of both MoNCD and skill mix on nurse outcomes (i.e., RN job stress and role tension; McGillis Hall & Doran, 2007). Skill mix was operationalized in two ways: as a continuous variable (the proportion of regulated nurses to unregulated staff) and as a categorical variable (all-RN, RNs or LPNs, RNs or LPNs or care aides). Mode was operationalized as total patient care versus other (i.e., team nursing and primary nursing). Skill mix was not related to job stress outcomes. However, compared with other MoNCDs, total patient care was associated with lower job stress (McGillis Hall & Doran, 2007). A key limitation of this study is its lack of control for conditions of nurses’ work environments and workload factors.

Researchers in four other studies examined only the effect of MoNCD on nurse outcomes. In two quasi-experimental studies, researchers compared RNs’ satisfaction scores after total patient care was replaced with team nursing and found no statistically significant differences after this change (Lee, Yeh, Chen, & Lien, 2005; Tran, Johnson, Fernandez, & Jones, 2010). In both studies, a small sample was used (*N* ≤ 38). Similarly, Huang et al. (2011) examined the change in satisfaction of only 38 RNs in a cross-sectional study after total patient care was replaced with team nursing and found no significant changes in RNs’ satisfaction scores. Researchers in a Canadian longitudinal study (Wells, Manuel, & Cunning, 2011) who examined the change in nurse job satisfaction and empowerment after team nursing was replaced with total patient care across three time periods (*N* = 38, 36, and 21 at Times 1, 2, and 3) reported that no changes were detected across the three time periods. Overall, researchers in all four studies failed to find a relationship between MoNCD and nurse outcomes which may be due to their small sample size.

### Conceptual Framework

**Figure 1.**
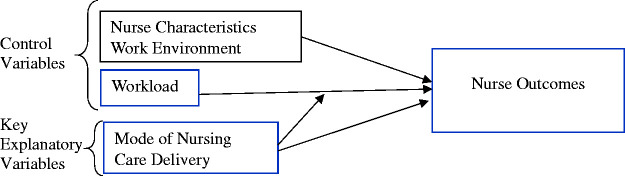
Conceptual model.

The conceptual framework for this study (Figure 1) is based on a specific portion of an evidence-based model, the Nursing Worklife Model (Lake, 2002). The Nursing Worklife Model components, particularly “the nursing model of care delivery” and “staffing and resource adequacy” components, were previously linked to improved nurse and patient outcomes (Laschinger, 2008; Laschinger & Leiter, 2006). Lake (2002) described nursing model of care delivery as hospitals supporting a nursing model rather than a medical model of care. In this study, the “nursing model of care delivery” component is operationalized as the MoNCD and skill mix. Similarly, the “staffing and resource adequacy” component is a proxy for nurses’ perceptions of workload management (Lake, 2002). Previous research supports the relationship between other control variables, including nurse characteristics and work environment factors, and nurse outcomes (Hayes et al., 2012; Warshawsky & Havens, 2011).

### Purpose

The purpose of this study is to examine the relationship between two key models of care delivery components, MoNCD and skill mix, and nurse outcomes. The study design was specifically chosen to address the limitations noted in previous studies by examining the effect of both MoNCD and skill mix on nurse outcomes; differentiating between the two most common MoNCDs (i.e., total patient care and team nursing); including an adequate sample size; and controlling for the effect of known predictors of nurse outcomes such as nurse characteristics, workload factors, and work environment factors.

The three key research questions are as follows:

1. What is the relationship between care delivery model components, MoNCD and skill mix, and nurse outcomes after controlling for nurse characteristics, nurse workload factors, and work environment factors?
2. Does MoNCD moderate the relationship between nurse workload factors and nurse outcomes?
3. Does skill mix moderate the relationship between nurse workload factors and nurse outcomes?

## Methods

### Design

This was an exploratory cross-sectional correlational survey study based on a secondary analysis of data. The larger study examined the impact of nurses’ workload on nurse and patient outcomes. A detailed description of the larger study design can be found in MacPhee, Dahinten, & Havaei (2017). The larger study used a proportionate stratiﬁed sampling strategy based on health authorities and employment status. A total of 15,702 acute care nurses were randomly selected from the BC nurses’ union database and received postcards with unique passwords, inviting them to complete a web-based survey. E-mail reminders were sent at 2-week intervals over a month. A second invitation, consisting of a paper version of the survey, was mailed out to a random subset of 1,500 nurses. Respondents were informed of the voluntary nature of their participation and the anonymity of their responses.

The inclusion criteria for the current study included direct care RNs with a practicing status and working in medical or surgical specialties. This resulted in a sample size of 416 RNs. It is unclear how many medical–surgical nurses were invited to participate in the larger study; hence, a response rate could not be computed. A priori power calculation showed a sample size of about 226 individuals would have sufficient power to detect small effect sizes (i.e., *R*^2 ^= .10) for multiple regression analysis at alpha = .05, and with about 20 predictors.

### Measurement

Survey items were based on the RN4CAST, an international study of organizational characteristics of hospital care impact on nurse recruitment, nurse retention, and patient outcomes. The RN4CAST survey has received rigorous psychometric testing, and it has been used in nursing workforce research in 12 European countries and the United States (Sermeus et al., 2011). A more detailed description of study measures can be found in Havaei et al. (2019).

#### Outcome variables

Two nurse outcome variables included (a) emotional exhaustion and (b) job satisfaction. Emotional exhaustion was measured with the emotional exhaustion subscale of the Maslach Burnout Inventory-Human Service Scale (Schaufeli, Leiter, & Maslach, 2009). This 9-item measure asked nurses to rate their feelings of psychological depletion due to work burden on a 7-point scale (0 = *never* to 6 = *daily*; Schaufeli et al., 2009). Sum scores ranged from 0 to 54. A principal component analysis with varimax rotation among the study sample confirmed a unidimensional factor structure with factor loadings ranging from .70 to .89 explaining 65% of the variance; Cronbach’s alpha was .93, demonstrating a satisfactory internal consistency.

Job satisfaction was measured with three items: (a) satisfaction with current job (1 = *very dissatisfied*, 4 = *very satisfied*), (b) intent to leave over the next year (1 = *very unlikely*, 4 = *very likely*), and (c) likelihood to recommend hospital to nursing colleagues as a place to work (1 = *definitely no*, 4 = *definitely yes*; Sermeus et al., 2011). Sum scores were computed after the intent to leave item was reverse coded. Possible sum scores ranged from 3 to 12 with higher scores indicating higher levels of job satisfaction. A principal component analysis with varimax rotation among the study sample confirmed a unidimensional factor structure with factor loadings of .61 to .87 explaining 50% of the variance; Cronbach’s alpha was .64 which is an acceptable internal consistency for scales with few items (Paul, 2000).

#### Key independent variables

A single item that asked nurses to endorse the option that best described how care was delivered in their primary unit over the last shift was used to measure MoNCD; response options included (a) patients were assigned to one nurse (e.g., total patient care, labeled as TPC) and (b) patients were assigned to a nursing team (e.g., team nursing, labeled as TN) (Havaei et al., 2019). These descriptions were based on Duffield et al. (2010) and confirmed by subject matter experts (i.e., professional practice officers of BC health authorities, senior nurse leaders from the provincial nurses’ union; 0 = TPC and 1 = TN).

Skill mix type was measured with an item that inquired about the number of each nurse type providing direct care in respondent’s primary unit (Havaei et al., 2019). Nurse types included RNs (or registered psychiatric nurses), LPNs, and unlicensed personnel (e.g., care aides). This information was used to identify skill mix type: (a) a skill mix that does not include LPNs (i.e., all-RN, and RNs or care aides) and (b) a skill mix that includes LPNs (i.e., RNs or LPNs, and RNs or LPNs or care aides; 0 = *a skill mix without LPNs*; 1 = *a skill mix with LPNs*).

#### Control variables

Nurse workload factors encompassed nurse staffing levels, patient acuity, and dependency. Nurse staffing levels reflected patient–RN ratios and patient–regulated nurse ratios (Sochalski, 2001). These ratios were computed using two questions that asked about the total number of patients and the total number of direct care nurses in the unit. The other two components of nurse workload were measured by questions based on the American Association of Critical Care Nurses’ Synergy Model (Curley, 2007). Nurses were asked to rate their patients’ overall levels of acuity (0 = *not acute at all*, 3 = *very acute*) and dependency (0 = *completely independent*, 3 = *completely dependent*) over the last month; acuity and dependency were recoded into binary variables (Havaei et al., 2019).

The Practice Environment Scale-Nursing Work Index (PES-NWI) was used to measure the quality of nurses’ work environments (Lake, 2002). For this study, a 28-item version of PES-NWI, consisting of five subscales, was used: (a) staffing and resource adequacy, (b) nurse-medical doctor (MD) relation, (c) nursing leadership, (d) participation in hospital affairs, and (e) nursing foundation of care delivery (Havaei et al., 2019). The items were rated on a 4-point scale (1 = *strongly disagree*, 4 = *strongly agree*) with higher mean subscale scores indicating a higher quality environment. Confirmatory factor analysis of the measure with the study sample confirmed a 5-factor model with a mediocre fit (root mean square error of approximation = .08, standardized root mean square residual = .07, goodness of fit index = .83, comparative fit index = .94, and normed fit index = .92); subscale Cronbach’s alphas ranged from .76 to .82 (Havaei et al., 2019).

Demographic questions included nurse characteristics such as age, gender (0 = *male*, 1 = *female*), nursing education (0 = *diploma*, 1 = *BSN or Masters*), years of nursing experience, employment status (0 = *full-time*, 1 = *part-time or casual*), employment contract (0 = *permanent*, 1 = *temporary*), and number of nursing jobs (0 = *one job*, 1 = *more than one job*) (Havaei et al., 2019).

### Statistical Analysis

Key methods of data analysis were chi-square analyses, hierarchical multiple regression, and moderated regression. Hierarchical multiple regression is used to identify if key predictors explain a statistically significant amount of variance in the dependent variable after accounting for control variables. Three multiple regression models were obtained to examine the relationships between key predictors, MoNCD and skill mix, and each outcome (Research Question 1). Control variables including nurse characteristics, nurse workload, and work environment factors were entered into the first model followed by MoNCD and skill mix in the second and third models, respectively. Four interaction terms between MoNCD and workload factors (Research Question 2) and four interaction terms between skill mix and workload factors (Research Question 3) were obtained; each interaction term was examined separately and entered into the fourth regression model after skill mix. To reduce the effects of multicollinearity in moderated regression, continuous predictors were standardized prior to being introduced into the regression analyses (Dawson, 2014). To maximize power, only significant interaction terms were retained in the final models.

## Results

The majority of participants (97%) were female with a mean age of 38.3 years. About three quarters of participants were bachelors prepared with permanent contracts. Over half of the sample were full time compared with 24% with part time and 20% with casual employment status. About 82% of the sample had one nursing job compared with 17% who had two or more jobs.

The descriptive statistics on key study variables are shown in Table 1. About 80% of participants identified their patients as moderately or very acute. More than 85% of participants identified their patients as somewhat or very dependent. On average, participants reported patient–nurse ratios as 7:1 patients per RN and 4:1 patients per regulated nurse. Three of the five work environment factors were scored more favorably: nurse-MD relation, nursing foundation of care delivery, and nursing leadership. More than three quarters of nurses identified their MoNCD as TPC compared with one quarter who reported providing care based on TN. With respect to skill mix, over two thirds of nurses reported working with LPNs as opposed to about one third who reported working without LPNs. Mean emotional exhaustion scores were high (*Mean* = 27, *SD* = 12.9; Silva et al., 2015). Mean job satisfaction scores were about 8 (*SD* = 2.1). The relationships between study variables are shown in Table 2.

**Table 1.** Descriptive Statistics of the Study Key Variables (*N* = 416).

Characteristics	*f* (%)	*M* (*SD*)	*Range*
Patient acuity			
Not at all acute	5 (1.2%)	–	–
Somewhat acute	76 (18.4%)	–	–
Moderately acute	236 (57.3%)	–	–
Very acute	95 (23.1%)	–	–
Patient dependency			
Very independent	18 (4.4%)	–	–
Somewhat independent	41 (10.0%)	–	–
Somewhat dependent	202 (49.1%)	–	–
Very dependent	150 (36.5%)	–	–
Patient–nurse ratios			
Patient–RN	–	6.6 (4.3)	.3–34
Patient–regulated nurse	–	4.4 (1.8)	.3–11.2
Nursing work index subscales			
Staffing and resource adequacy	–	2.1 (.6)	1–4
Nurse-MD relation	–	2.8 (.5)	1–4
Nursing leadership	–	2.4 (.6)	1–4
Participation in hospital affairs	–	2.1 (.5)	1–3.5
Nursing foundation of care delivery	–	2.6 (.4)	1–4
Mode of nursing care delivery			
Total patient care	320 (76.9%)	–	–
Team nursing	96 (23.1%)	–	–
Skill mix			
Without LPNs (i.e., All-RNs or RNs or CAs)	133 (32.0%)	–	–
With LPNs (i.e., RNs or LPNs or RNs or LPNs or CAs)	283 (68.0 %)	–	–
Emotional exhaustion	–	27.3 (12.9)	0–54
Job satisfaction	–	7.7 (2.1)	3–12

*Note.* RNs = registered nurses; LPNs = licensed practical nurses; CAs = care aides.

**Table 2.** Correlations Between Key Study Variables (*N* = 416).

Variable	1	2	3	4	5	6	7	8	9	10	11	12	13	14	15	16	17	18	19
1. Age	–																		
2. Gender	**−.10**	–																	
3. Education	**−.70**	.05	–																
4. Nursing experience	**.86**	**−**.03	**−.70**	–															
5. Employment status	**−.13**	.05	.05	**−.10**	–														
6. Contract	**−.20**	**−**.05	**.15**	**−.18**	**.50**	–													
7. Nursing jobs	**−.10**	.04	**.12**	**−**.09	**.22**	**.20**	–												
8. Patient acuity	**−**.05	**−**.04	.02	**−**.05	**−**.05	**−**.08	**−.13**	–											
9. Patient dependency	**−**.08	.07	.06	**−**.09	**−**.08	**−**.02	**−**.05	**.11**	–										
10. Pt-RN ratios	.02	.05	.00	.01	**−**.08	.03	.04	**−.12**	.03	–									
11. Pt-Regulated nurse ratios	**−**.01	.03	.01	**−**.01	**−**.01	.06	.08	**−.13**	.00	**.73**	–								
12. Staffing and resource adequacy	**−**.03	.05	.04	.00	.06	.05	.03	**−.24**	**−.20**	**−.17**	**−.11**	–							
13. Nurse-MD relation	.03	**−**.07	.04	**−**.01	.03	.01	**−**.01	**−**.05	**−**.05	**−**.02	.02	**.28**	–						
14. Nursing leadership	**−.15**	.03	**.19**	**−.18**	**.11**	**.11**	.04	**−.15**	**−.15**	**−**.05	**−**.02	**.52**	**.34**	**–**					
15. Participation in hospital affairs	**−.15**	.07	.10	**−.16**	**.15**	**.13**	**−**.01	**−.12**	**−**.05	**−.16**	**−**.07	**.53**	**.28**	**.66**	–				
16. Nursing foundation of care	**−**.06	**.11**	.07	**−.11**	.09	.05	**−**.06	**−.13**	**−.11**	**−.16**	**−**.07	**.58**	**.38**	**.60**	**.66**	–			
17. Mode of nursing care delivery	**.10**	.05	**−**.08	**.14**	.01	**−**.01	.03	.01	**−**.07	**.20**	.05	**−**.09	**−**.08	**−**.05	**−.20**	**−.16**	–		
18. Skill mix	**−.10**	**−**.02	**.13**	**−.11**	.03	.02	.00	.05	**.12**	**.36**	.05	**−.19**	**−**.01	**−**.08	**−**.08	**−.14**	.05	–	
19. Emotional exhaustion	**−**.04	**−**.01	**−**.02	**−**.03	**−.17**	**−.14**	**−**.04	**.22**	.07	.07	.01	**−.53**	**−.17**	**−.40**	**−.44**	**−.37**	.05	.09	–
20. Job satisfaction	.01	**−**.03	.03	.01	**−**.01	**−**.04	**−**.03	**−.15**	.03	**−**.09	**−**.09	**.48**	**.25**	**.38**	**.43**	**.44**	**−.17**	**−**.04	**−.58**

*Note.* RNs = registered nurses.

*p* < .05 correlations are boldface.

Chi-square analyses showed no statistically significant differences in skill mix between nurses who identified their MoNCD as TPC versus TN (χ^2 ^= .36, *p* > .05). Among nurses who worked in a TPC MoNCD, 67% identified their skill mix as including LPNs, whereas 72% of nurses who worked in TN MoNCD identified their skill mix as including LPNs.

### Hierarchical Multiple Regression Findings

With the exception of age, staffing and resource adequacy, participation in hospital affairs, and the interaction term, Acuity × Skill Mix, no other variables were related to emotional exhaustion (Table 3). Overall, there were no statistically significant changes in *R*^2^ between Models 1, 2, and 3, but the *R*^2^ increased by 1.1% from Model 3 to Model 4 after the addition of Acuity × Skill Mix. The final model, Model 4, explained 38% of the variance in emotional exhaustion scores, *F*(19, 295) = 9.41, *p < *.001.

**Table 3.** Hierarchical Regression Analysis Results for Variables Predicting Nurse Outcomes (*N* = 416).

	Emotional exhaustion Model 4				Job satisfaction Model 3			
	β	CI (95%)	*p*	*R* ^2^	β	CI (95%)	*p*	*R* ^2^
				37.7				35.0
Age	−.22^*^	[−5.53, −.21]	.03		.05	[−.34, .55]	.65	
Gender	.03	[−5.48, 9.41]	.60		−.05	[−1.84, .64]	.34	
Education	−.08	[−6.69, 1.97]	.28		.03	[−.58, .86]	.70	
Nursing experience	.03	[−2.13, 3.03]	.73		.03	[−.38, .49]	.80	
Employment status	−.09	[−5.18, .62]	.12		−.03	[−.62, .34]	.57	
Contract	−.07	[−5.52, 1.04]	.18		−.10	[−1.04, .04]	.07	
Nursing jobs	.01	[−2.89, 3.81]	.79		.00	[−.53, .57]	.95	
Acuity	.20^*^	[1.32, 12.85]	.02		−.05	[−.86, .26]	.29	
Dependency	−.09	[−6.75, .29]	.07		.12^*^	[.16, 1.35]	.01	
Patient–RN	.04	[−3.02, 5.31]	.59		.08	[−.32, 1.06]	.29	
Patient–regulated nurse	−.08	[−5.46, 1.43]	.25		−.09	[−.94, .20]	.21	
Staffing and resource adequacy	−.40^***^	[−7.11, −3.80]	.00		.25^***^	[.29, .84]	.00	
Nurse–MD relation	.02	[−1.04, 1.59]	.68		.08	[−.03, .40]	.10	
Nursing leadership	−.10	[−3.07, .45]	.15		.07	[−.15, .44]	.33	
Participation in hospital affairs	−.19^**^	[−4.23, −.66]	.01		.19^**^	[.12, .71]	.01	
Nursing foundation of care	.00	[−1.81, 1.86]	.98		.15^*^	[.03, .64]	.03	
Mode of nursing care delivery	−.01	[−3.36, 2.73]	.84		−.06	[−.84, .18]	.21	
Skill mix	.27^*^	[.92, 14.11]	.03		−.02	[−.59, .42]	.74	
Acuity × Skill Mix	−.31^*^	[−15.14, −1.08]	.02		–	–	–	
*F*(*df*1, *df*2)	*F*(19, 295) = 9.41^***^				*F*(18, 295) = 8.84^***^			

*Note.* β = standardized beta coefficient; 95% CI = 95% confidence intervals; RNs = registered nurses.

The results are for the final regression models.

* *p* < .05. **p < .01. ***p < .001.

**Figure 2.**
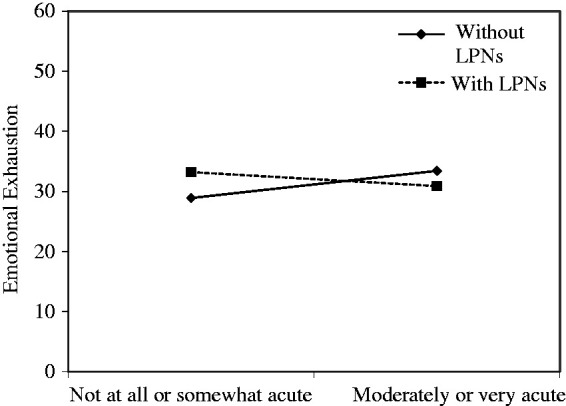
The moderating effect of skill mix on the relationship between patient acuity and nurse emotional exhaustion.

With the exception of patient acuity and skill mix, other significant variables were negatively related to emotional exhaustion. Among primary effects, staffing and resource adequacy (β = −.40, *p* < .001) and skill mix (β = .27, *p* < .05) were the strongest predictors of emotional exhaustion. The negative beta associated with staffing and resource adequacy suggests that one standard deviation increase in staffing and resource adequacy would result in a .40 standard deviation decrease in emotional exhaustion. The interaction term between patient acuity and skill mix was found to be statistically significant (β = −.31, *p* < .05), thus indicating that skill mix moderated the relationship between patient acuity and nurse emotional exhaustion. At higher levels of acuity, nurses who worked with LPNs reported lower levels of emotional exhaustion than their peers who worked without LPNs (see Figure 2).

With the exception of patient dependency, staffing and resource adequacy, participation in hospital factors, and nursing foundation of care delivery, none of the other variables were related to job satisfaction scores (Table 3). None of the interaction terms were significantly related to job satisfaction and hence were dropped from the regression model. There were no statistically significant changes in *R*^2^ between Models 1, 2, and 3. Overall, the final model explained 35% of variance in job satisfaction, *F*(18, 295) = 8.84., *p < *.001.

All significant variables were positively related to job satisfaction. The two strongest predictors were staffing and resource adequacy (β = .25, *p* < .001) and participation in hospital affairs (β = .19, *p* < .01). The positive beta coefficients suggest that one standard deviation increase in staffing and resource adequacy and participation in hospital affairs would result in .25 and .19 standard deviation increase in job satisfaction, respectively. Patient dependency was also positively related to job satisfaction (β = .12, *p* < .05), which suggests that a one standard deviation increase in patient dependency would result in a .12 standard deviation increase in job satisfaction. The lack of a significant interaction term suggests that MoNCD and skill mix did not moderate the relationship between workload factors and job satisfaction.

## Discussion

Overall, there were four key findings: (a) There were no differences in skill mix between total patient care and team nursing, (b) skill mix moderated the relationship between patient acuity and emotional exhaustion, (c) MoNCD was not related to nurse outcomes, and (d) certain aspects of nurses’ work environments were the most important predictors of both nurse outcomes.

Contrary to institutional reports of transition from total patient care to team nursing in acute care settings (British Columbia Ministry of Health, 2014), only a small proportion of participants reported team-based care provision. This finding may suggest that transition from total patient care to team nursing in BC medical–surgical settings has been slower than anticipated because of inadequate sustained organizational supports and resources that facilitate this transition.

A majority of RNs who practiced total patient care reported working in a skill mix including LPNs. This finding is unexpected as total patient care has been traditionally associated with an all-RN skill mix (Duffield et al., 2010; Shirey, 2008) and may signal that there are LPNs who have their own independent patient assignments in BC medical–surgical settings. The larger study data with LPNs revealed that 59% of medical–surgical LPNs reported total patient care as their MoNCD, and 61% of LPNs provided care to moderately or very acute patients. Questions are raised, therefore, about LPNs’ working beyond their scope of practice. This speculation is consistent with recent research evidence that found some LPNs and a majority of RNs from remote and rural settings performed nursing competencies beyond their legal scope of practice (MacLeod et al., 2019a, 2019b). In BC, although LPNs are self-regulated, they only care for stable, less acute patients.

Contrary to RNs working without LPNs, RNs working with LPNs reported lower levels of emotional exhaustion when providing care for higher acuity patients. This finding could be attributed to RNs valuing the support of their LPN colleagues especially at times of high workload. Previous research shows coworker support was related to the three dimensions of burnout (i.e., emotional exhaustion, depersonalization, and personal accomplishment). A cross-sectional survey study of 210 Spanish nurses found higher collegial support was associated with lower emotional exhaustion scores (Albar Marín & Garcia-Ramirez, 2005). More specifically, compared with other types of support (from kin and supervisor), collegial support was identified as the most important predictor of this dimension of burnout. Similarly, a cross-sectional study of 1,561 Swedish RNs and nursing assistants found, compared with other types of support (e.g., supervisory support), coworkers’ support was the most important predictor of all burnout dimensions (Sundin, Bildt, Lisspers, Hochwalder, & Setterlind, 2006). Thus, the presence of LPNs may have provided RNs with higher collegial support perceptions which subsequently protected them from developing emotional exhaustion when caring for higher acuity patients.

MoNCD was not related to nurse outcomes. This finding can be explained by the unexpectedly strong effects of some of the control variables on these study outcomes. In particular, work environment factors were the strongest predictors of both outcomes. For example, the strongest predictor of both nurse outcomes was the staffing and resource adequacy component of the work environment. This finding means MoNCD was not related to nurse outcomes over and above the effect of other control variables in particular work environment factors.

Similar to earlier studies (Laschinger, 2008), nursing work environment factors, particularly staffing and resource adequacy, participation in hospital affairs, and nursing foundation of care delivery, were the most important predictors of nurse outcomes. Among PES-NWI studies, only one study was located in which all five PES-NWI subscales were examined using multiple regression analysis (Hessels, Flynn, Cimiotti, Cadmus, & Gershon, 2015). This study found large effect sizes for the three work environment factors and nursing tasks left undone (β = .47–.77).

### Implications for Practice

The study findings have implications for research, policy, and practice. First, given the importance of work environment factors, we recommend nursing leaders and policy makers to invest in those workplace conditions that improve nurse outcomes. In particular, sufficient staffing and resources and opportunities for nurse participation in organizational affairs were found to be important to nurses. Second, as a skill mix with LPNs was found to buffer against the negative effects of high workload on RN emotional exhaustion, we believe nurse leaders can use skill mix considerations as a strategy to enhance nurse outcomes. That said, future research should also include LPNs’ perspectives; the effect of skill mix and MoNCD should be examined on both RNs and LPNs outcomes across a variety of acute care settings. Third, this study found some LPNs may be caring for high acuity patients independently; this finding raises red flags about LPNs’ adherence to their scopes of practice. Accordingly, we strongly recommend future research to investigate LPNs’ scope of practice in light of the workplace MoNCDs. Finally, the study findings suggested a slow transition from total patient to team nursing across BC medical–surgical settings. At this time, there are no province-wide data available on the extent to which team-based MoNCDs are utilized in BC acute care settings. Policy makers and researchers should work together to gain a deeper understanding of the extent to which team-based care delivery is utilized in provincial acute care settings.

### Strength and Limitation

To our knowledge, this is the first study to examine the effect of both MoNC and skill mix on Canadian medical–surgical RN outcomes. But the study findings should be interpreted with considerations of its limitations. First, no cause and effect conclusions can be made due to the cross-sectional design of the study. Also, for reasons of confidentiality, individual nurses were not linked to particular units; however, disaggregation of data may increase the likelihood of making a Type I error as the between-group variance is ignored in such circumstances (Woltman, Feldstain, MacKay, & Rocchi, 2012). Another limitation is the low response rate of the larger study that leads to concerns of sample bias and generalizability of the findings. To increase response rate, several strategies were implemented in the larger study: advertisements through union media, e-mail reminders to nonrespondents, incentives, and hard copy send-outs to a random sample of nurses. Our study sample demographics, however, were similar to the BC nursing workforce with respect to age, gender, and employment status (Canadian Institute for Health Information, 2010). Also, because some of the study measures relied on nurse reports of phenomena that occurred in the past, there is a possibility of measurement error attributed to recall bias. Ideally, nurse self-reports of quality and safety status should be compared with administrative data. Unfortunately, access to these data is currently limited in BC. But, research shows self-reports are a useful substitute in circumstances where administrative data are not available (McHugh & Stimpfel, 2012).

## Conclusions

In sum, there were four key findings in this study: (a) Work environment factors were the strongest predictors of nurse outcomes, highlighting the need to attend to nurses’ working conditions; (b) a skill mix including LPNs was found to buffer against the negative effects of high workload on RN emotional exhaustion; (c) some BC medical–surgical LPNs provided total patient care to high acuity patients; and (d) there has been a slow transition from total patient care to team nursing in most medical–surgical settings. Ultimately, improving nurse outcomes is dependent on several factors. A key factor is a flexible MoNCD that is determined by unit-level nursing human resources and their competencies. Research, policy, and best practices must ensure that nurses with the right competencies adopt the most appropriate approach to care (MoNCD) to address patients’ needs.
